# Correction: Tsuneki et al. Deep Learning-Based Screening of Urothelial Carcinoma in Whole Slide Images of Liquid-Based Cytology Urine Specimens. *Cancers* 2023, *15*, 226

**DOI:** 10.3390/cancers16244277

**Published:** 2024-12-23

**Authors:** Masayuki Tsuneki, Makoto Abe, Fahdi Kanavati

**Affiliations:** 1 Medmain Research, Medmain Inc., Fukuoka 810-0042, Japan; 2 Department of Pathology, Tochigi Cancer Center, 4-9-13, Yohnan, Utsunomiya 320-0834, Japan

There were errors in the original publication [1]. The authors used SurePath liquid-based cytology (LBC) specimens, not ThinPrep. The corrected statements are as follows:

Name of section: 1. Introduction

Paragraph number: 4

“In this study, we trained deep learning models based on convolutional neural networks (CNN) using a training dataset of 786 urine LBC (SurePath) WSIs.”

Name of section: 2. Materials and Methods

Name of sub-section: 2.1. Clinical Cases and Cytopathological Records

Paragraph number: 1

“In this retrospective study, a total of 1556 LBC SurePath (Becton Dickinson) conventionally prepared cytopathological slide glass specimens of human urine cytology were collected from a private clinical laboratory in Japan after routine cytopathological review of those glass slides by cytoscreeners and pathologists.”

“We have confirmed that cytoscreeners and pathologists were able to classify (Table 1) from the visual inspection of the LBC SurePath (Becton Dickinson) stained WSIs alone.”

Name of section: 4. Discussion

Paragraph number: 3

“One limitation of this study is that it primarily included urine LBC (SurePath) WSIs (both training and test sets) from a single private clinical laboratory in Japan.”

“Validations on a wide variety of specimens from multiple different origins (both clinical laboratories and hospitals) and other LBC method(s) (e.g., ThinPrep) would be essential for ensuring the robustness of the models.”

The authors apologize for any inconvenience caused and state that the scientific conclusions are unaffected. This correction was approved by the Academic Editor. The original publication has also been updated.
